# Prediction of performance in a 100-km run from a simple equation

**DOI:** 10.1371/journal.pone.0279662

**Published:** 2023-03-02

**Authors:** Jeremy B. Coquart

**Affiliations:** Univ. Lille, Univ. Artois, Univ. Littoral Côte d’Opale, ULR 7369—URePSSS—Unité de Recherche Pluridisciplinaire Sport Santé Société, Lille, France; Shenzhen Baoan Women’s and Children’s Hospital, CHINA

## Abstract

This study aimed to identify predictive variables of performance for a 100-km race (Perf_100-km_) and develop an equation for predicting this performance using individual data, recent marathon performance (Perf_marathon_), and environmental conditions at the start of the 100-km race. All runners who had performed official Perf_marathon_ and Perf_100-km_ in France, both in 2019, were recruited. For each runner, gender, weight, height, body mass index (BMI), age, the personal marathon record (PR_marathon_), date of the Perf_marathon_ and Perf_100-km_, and environmental conditions during the 100-km race (*i*.*e*., minimal and maximal air temperatures, wind speed, total amount of precipitation, relative humidity and barometric pressure) were collected. Correlations between the data were examined, and prediction equations were then developed using stepwise multiple linear regression analyses. Significant bivariate correlations were found between Perf_marathon_ (*p*<0.001, *r* = 0.838), wind speed (*p*<0.001, *r* = -0.545), barometric pressure (*p*<0.001, *r* = 0.535), age (*p* = 0.034, *r* = 0.246), BMI (*p* = 0.034, *r* = 0.245), PR_marathon_ (*p* = 0.065, *r* = 0.204) and Perf_100-km_ in 56 athletes The, 2 prediction equations with larger sample (n = 591) were developed to predict Perf_100-km_, one including Perf_marathon_, wind speed and PR_marathon_ (model 1, *r²* = 0.549; standard errors of the estimate, SEE = 13.2%), and the other including only Perf_marathon_ and PR_marathon_ (model 2, *r²* = 0.494; SEE = 14.0%). Perf_100-km_ can be predicted with an acceptable level of accuracy from only recent Perf_marathon_ and PR_marathon_, in amateur athletes who want to perform a 100 km for the first time.

## Introduction

The popularity of the ultramarathon has increased tremendously over the last decades, with increasingly more organized events every year [1, 2]. An ultramarathon is currently defined as any running event taking longer than 6 hours [3]. Ultramarathon races are generally held as time-limited events (e.g., 24-hour races) or distance-limited events, such as the 100-km races [4].

The ability to predict running performance is of great interest for athletes and coaches, particularly in the ultramarathon. Indeed, it is helpful for prescribing speeds during tempo runs, determining the optimal pace strategy during the race, and even choosing splitting times [5–7].

Knechtle et al. proposed a simple equation to predict performance in 24-hour [8] and 100-km [9] races. These authors showed that performance in the 100-km race (Perf_100-km_) is primarily related to training intensity and volume, as well as to the age of the runners, but less so to their anthropometric characteristics [9]. However, this study was based only on data from male ultramarathoners at the Biel ultramarathon in Switzerland, and the particular conditions of this race (start at 10:00 p.m., first weekend of June, no rain…) make it difficult to generalize these results to other races, especially in France where 100-km races are usually held in the daytime in September/October with generally lower temperatures. Indeed, temperature and thus season have been shown to influence marathon performance in [10–13].

The authors [9] were also able to obtain an important indicator: the personal best in the marathon. However, this record may have been achieved years earlier (or even decades) and, although it was associated with Perf_100-km_ from bivariate correlation analysis (*p* < 0.0001, *r* = 0.65), this potential predictive variable was excluded from stepwise multiple-regression analysis [9]. Indeed, an old record may sometimes no longer be representative of a runner’s current marathon performance potential and thus of 100-km performance. Therefore, a recent marathon performance (within the last 9 months) may be more appropriate.

The aim of the current study was to identify the predictive variables of Perf_100-km_ and develop an equation for predicting performance, using individual data, a recent marathon performance, and the environmental conditions at the start of the 100-km race.

## Materials and methods

### Procedure

All French official rankings of the French Athletics Federation (FFA for *Fédération Française d’Athlétisme*) in 2019 for the marathon (*n* = 88,455) and the 100-km run (*n* = 1,560) were retrospectively scrutinized. Only French competitions have been selected. From these rankings, all athletes who had competed in both were retained (*n* = 591). Then, runners who had not self-reported their weight and/or height and/or birth date were removed from the analysis (*n* = 533). Thus, 58 athletes were included in this stage. Moreover, runners who maintained a higher speed in the 100-km run than in the marathon were also removed (*n* = 2). Therefore, 56 athletes were ultimately included in the statistical analysis.

For each athlete, gender (*i*.*e*., woman *vs* man), birth date (to calculate the age), weight and height (to calculate the body mass index: BMI) were collected. Moreover, the race times on the 100-km run (*i*.*e*., Perf_100-km_) and the marathon (*i*.*e*., Perf_marathon_), attaining (or not) of personal record during the marathon (PR_marathon_), and the dates of participation in the marathon (*i*.*e*., Date_marathon_) and 100-km run (*i*.*e*., Date_100-km_) were recorded, these last in order to determine the moment of the performances (*i*.*e*., the number of days since January 1, 2019) and to calculate the interval between the performances in the marathon and the 100-km. Last, for each 100-km race, city, Date_100-km_, minimal and maximal air temperatures, wind speed, total amount of precipitation, relative humidity and barometric pressure were collected.

This study was approved by the National Ethics Committee for Research in Sports Sciences (CERSTAPS 2019-22-02-31). Moreover, the protocol for this study was legally declared, in accordance with the European General Data Protection Regulations.

### Statistical analysis

Standard statistical methods were used to calculate the means and standard deviations (SD).

Pearson product-moment correlations were used to evaluate the bivariate associations between dependent (*i*.*e*., Perf_100-km_) and independent variables (*i*.*e*., Perf_marathon_, gender, age, weight, height, BMI, PR_marathon_, Date_marathon_, Date_100-km_, interval between performances, minimal and maximal air temperatures, wind speed, total amount of precipitation, relative humidity and barometric pressure).

Then, two prediction equations were developed from only the significantly correlated variables (*p* < 0.10) using stepwise multiple linear regression analysis. The first multiple linear regression analysis included all variables correlated with Perf_100-km_, while the second analysis included the characteristics of the athletes and their marathon performance but excluded the environmental conditions of the 100-km (*i*.*e*., minimal and maximal air temperatures, wind speed, total amount of precipitation, relative humidity and barometric pressure), since it is difficult to predict them accurately well in advance of the race.

The variance inflation factor (VIF) was used to detect the severity of multicollinearity among the independent variables in the regression models.

Fisher’s tests were used to examine the contribution of each variable in the two models, and the results were confirmed by the analysis of the standardized β coefficients.

Moreover, the relationship between the Perf_100-km_ estimated by the prediction equation and actual Perf_100-km_ was analyzed with the Bravais-Pearson method and quantified with Pearson’s correlation coefficient. The 95% confidence interval (95%CI) were also calculated.

The standard error of the estimate (SEE) and percentage of SEE were calculated to establish the accuracy of the prediction equations.

Statistical significance was set at *p* < 0.05 and all analyses were performed with the SPSS package (release 20.0, Chicago, IL, USA).

## Results

The characteristics of the 56 runners are presented in Table 1.

**Table 1:** Characteristics of 56 athletes and their performances in marathon and 100-km.

	Mean ± SD	Effectif (percentage)	Range
Women	13 (23.2)		
Age (y)	48.7 ± 8.8		30–73
Weight (kg)	65.0 ± 8.3		43–83
Height (cm)	174 ± 8		154–193
BMI (kg.m^-2^)	21.5 ± 1.8		17.9–27.4
Perf_marathon_	3h28 ± 35 min		2h34-5h10
PR_marathon_	14 (25.0)		
Date_marathon_ (days after the january 1, 2019)	159 ± 83		61–327
Perf_100-km_	10h29 ± 2h16		7h13-17h20
Date_100-km_ (days after the january 1, 2019)	242 ± 70		102–284
Delay between marathon and 100-km run (days)	83 ± 118		13–223

From the sample of 56 subjects, significant bivariate correlations were found between Perf_marathon_ (*p* < 0.001, *r* = 0.838, 95%IC between 0.817 and 0.931), wind speed (*p* < 0.001, *r* = -0.545, 95%IC between -0.508 and -0.041), barometric pressure (*p* < 0.001, *r* = 0.535, 95%IC between 0.195 and 0.615), age (*p* = 0.034, *r* = 0.246, 95%IC between 0.277 and 0.666), BMI (*p* = 0.034, *r* = 0.245, 95%IC between 0.330 and 0.697), PR_marathon_ (*p* = 0.065, *r* = 0.204, 95%IC between -0.253 and 0.272) and Perf_100-km_. Therefore, only these variables were included in models, because they were significantly correlated to Perf_100-km_ with *p* < 0.10.

Nevertheless, the first stepwise multiple linear regression analysis entered only Perf_marathon_, wind speed and PR_marathon_ as the independent variables to yield the prediction equation, considering that the other variables (*i*.*e*., barometric pressure, age and BMI) were redundant with each other. Therefore, to improve the quality of the model (from a larger sample size; n = 591), subjects with missing variables were re-injected into the statistical analysis.

For this new statistical analysis, the characteristics of these 591 runners and environmental conditions are presented in Tables 2 and 3, respectively.

**Table 2:** Characteristics of 591 athletes and their performances in marathon and 100-km.

	Mean ± SD	Effectif (percentage)	Range
Women	99 (16.8)		
Perf_marathon_	3h52 ± 40 min		2h34-6h15
PR_marathon_	343 (58.0)		
Date_marathon_ (days after the january 1, 2019)	155 ± 87		26–348
Perf_100-km_	12h17 ± 2h25		6h48-21h30
Date_100-km_ (days after the january 1, 2019)	250 ± 58		102–284
Delay between marathon and 100-km run (days)	95 ± 112		6–258

**Table 3:** Characteristics and environmental conditions during the 100-km races.

City	Metz	Millau	Belves	Amiens
Day	07/09/2019	28/09/2019	13/04/2019	12/10/2019
Start time (h:min)	6h30	10h00	8h00	6h30
Mimimal air temperature (°C)	12	13	8	14
Maximal air temperature (°C)	17	19	15	19
Mean wind speed (m.s^-1^)	3.33	2.78	2.78	6.39
Total amount of precipitations (mm)	9	0	0	0
Mean relative humidity (%)	71	85	63	87
Mean barometric pressure (hPa)	1022	1022	1022	1011
First runner’s time (h:min)	8h13	7h27	6h55	6h49
Sample size (n)	47	324	76	144

From the sample of 591 subjects, significant bivariate correlations were found between Perf_marathon_ (*p* < 0.001, *r* = 0.696, 95% IC between 0.672 and 0.751), wind speed (*p* < 0.001, *r* = -0.394, 95%IC between -0.460 and -0.324), PR_marathon_ (*p* = 0.001, *r* = 0.127, 95%IC between 0.054 and 0.212) and Perf_100-km_.

The proposed prediction equation (*i*.*e*., model 1) is:

$${\text{Perf}}_{100-\text{km}}=265,512+2.335\times {\text{Perf}}_{\text{marathon}}-22,654\times \text{wind}\;\text{speed}+21.947\times {\text{PR}}_{\text{marathon}}$$ (model 1)

with Perf_100-km_ and Perf_marathon_ in minutes, wind speed in m.s^-1^ and PR_marathon_ = 1 when PR_marathon_ was performed or 0 when no PR_marathon_ has been performed.

Very low multicollinearity was found (because VIF < 5) for the independent variables (VIF < 1.069). The increase in *r*² from adding the second (*i*.*e*., wind speed) and third predictors (*i*.*e*., PR_marathon_) to the prediction equation was significant with F(1,588) = 76.225 (*p* < 0.001) and F(1,587) = 7.222 (*p* = 0.007), respectively. Moreover, Fisher’s test revealed a *p* < 0.001. The performance estimated by the prediction equation (including the three independent variables: Perf_marathon_, wind speed and PR_marathon_) was significantly correlated with the actual Perf_100-km_ (*r* = 0.741 and *r*² = 0.549). The standardized β coefficients and *p* values on Student’s t-test were 0.639 (*p* < 0.001), -0.241 (*p* < 0.001), and 0.075 (*p* = 0.007) for Perf_marathon_, wind speed and PR_marathon_, respectively. No autocorrelation in the residuals was noted. The 95%CI between actual and predicted Perf_100-km_ was between 0.719 and 0.788. The SEE for the prediction equation was 97 min, *i*.*e*., 13.2%.

The second multiple linear regression analysis (excluding environmental conditions) entered Perf_marathon_ and PR_marathon_ as the independent variables and yielded the following prediction equation (*i*.*e*., model 2):

$${\text{Perf}}_{100-\text{km}}=131.574+2.530\times {\text{Perf}}_{\text{marathon}}+30.113\times {\text{PR}}_{\text{marathon}}$$ (model 2)

with Perf_100-km_ and Perf_marathon_ in minutes and PR_marathon_ = 1 when PR_marathon_ was performed or 0 when no PR_marathon_ has been performed.

With VIF = 1.001 for both Perf_marathon_ and PR_marathon_, very low multicollinearity was found for the independent variables. The increase in *r*² from adding PR_marathon_ to the prediction equation was significant with F(2,588) = 12.335 (*p* < 0.001). The performance estimated by the prediction equation (including Perf_marathon_ and PR_marathon_) was significantly correlated with the actual Perf_100-km_ (*r* = 0.703 and *r*² = 0.494). The standardized β coefficients and *p* values on Student’s t-test were 0.692 (*p* < 0.001) and 0.103 (*p* < 0.001) for Perf_marathon_ and PR_marathon_, respectively. The analyse of the residuals indicated no autocorrelation. The 95%CI between actual and predicted Perf_100-km_ was between 0.680 and 0.758. The SEE for this second prediction equation was 103 min, *i*.*e*., 14.0%.

## Discussion

The current study aimed to identify the predictive variables of Perf_100-km_ in order to develop a prediction equation. The results showed significant bivariate correlations between Perf_100-km_ and individual data (*i*.*e*., age and BMI), recent performance and attaining (or not) of personal record during the marathon (*i*.*e*., Perf_marathon_ and PR_marathon_), and certain environmental conditions at the start of the 100-km race (*i*.*e*., wind speed and barometric pressure). However, only Perf_marathon_, PR_marathon_ and/or wind speed during the 100-km race were included in the prediction equations.

Age and BMI were significantly correlated with Perf_100-km_ in the bivariate correlation analysis, but these variables were removed from the multiple linear regression analyses because they were also significantly correlated with Perf_marathon_ (*p* < 0.001 and *r* = 0.433 for age, and *p* = 0.015 and *r* = 0.290 for BMI) and were thus predictive variables already included in prediction equations. This outcome is not surprising because Knechtle and colleagues showed that when the personal best Perf_marathon_ is included in the prediction equation, the addition of individual variables (*e*.*g*., BMI) does not improve the accuracy of the predicted time [9, 14, 15]. Moreover, age and BMI are known to be correlated with Perf_marathon_ [16].

In the present study, the barometric pressure during the 100-km races was negatively correlated with the wind, with a very high correlation coefficient (*p* < 0.001 and *r* = -0.998). Thus, when wind speed was entered into the stepwise multiple linear regression analysis, barometric pressure in the prediction equation was no longer related to Perf_100-km_ (*i*.*e*., model 1). Recent studies have confirmed the influence of wind speed on Perf_marathon_ [12, 13]. The current results confirm this for 100-km races; yet it would have been interesting to know the wind direction in order to determine whether it was a head wind, side wind or tail wind. In contrast to these studies [12, 13], a decrease in performance was nevertheless not found in races in the rain. Notably, of the four 100-km races included, only one experienced rainfall, and it involved only three athletes.

The prediction equation including wind speed during the 100-km race provided slightly more accurate predictions (SEE = 13.2 vs 14.0% for models 1 and 2, respectively). However, to allow athletes and/or coaches to predict Perf_100-km_ more simply, model 2 (without wind speed) may be sufficient. Indeed, predicting Perf_100-km_ to help the athlete determine the optimal pace strategy during the race and/or choose splitting times can be simple using a single previous Perf_marathon_ (in the last 9 months), whereas forecasting environmental conditions (often changing and difficult to forecast far in advance), such as wind speed, can be more complicated (for a low gain; *i*.*e*., improvement of 0.8% in accuracy). In the literature, about 10% accuracy is generally accepted as tolerable for predicting running performance [5], especially in amateur athletes who want to perform a 100 km for the first time.

It should be noted that, in the future, the accuracy of predictions might be improved by removing the limitations of the present study. For example, one limitation was the self-declaration of body height and weight and thus the calculation of athletes’ BMI. Height and weight were not measured in this study, but self-reported. Thus, the runners may have under- or overestimated these parameters. Nevertheless, it should be noted that runners are known to self-report their anthropometric data accurately [17]. Also, to avoid the possible influence of physical fitness between the marathon and the 100-km race, the two performances had to be performed within a time interval of 9 months. Yet, this time interval may not be negligible, thus allowing for significant changes in physical fitness. Similarly, although environmental conditions during the 100-km races were collected, this information was not available for the marathons. Therefore, it cannot be ruled out that some runners performed in different environmental conditions in the marathon and the 100-km race (*e*.*g*., marathon at 30°C in June *vs* 100-km at 15°C in September). Last but not least, running performance can be affected by a multitude of potential factors as physiological (*e*.*g*., maximal oxygen uptake, running economy, anaerobic threshold…), psychological (*e*.*g*., motivation, stress) and environmental (*e*.*g*., race profile: uphill, downhill…) variables. These variables have not been collected during the current study. However, a potential perceptive could be to include several of these variables to attempt to develop other models more accurate. However, despite these potential limitations (*i*.*e*., physical fitness, environmental conditions and psychological states between the marathon and the 100-km race), the proposed equations had an acceptable level of accuracy in amateur athletes. Nevertheless, a future study should confirm the validity of the 2 models presented in this study fom new sample of athletes.

## Conclusion

Perf_100-km_ was significantly correlated with individual data (*i*.*e*., age and BMI), recent performance and the attaining (or not) of personal record during the marathon (*i*.*e*., Perf_marathon_ and PR_marathon_), and certain environmental conditions at the start of the 100-km race (*i*.*e*., wind speed and barometric pressure). However, only Perf_marathon_, PR_marathon_ and wind speed during the 100-km race proved useful to predict Perf_100-km_. Moreover, for simplicity, model 2 including only Perf_marathon_ and PR_marathon_ (in the 9 months prior to a 100-km race) seems to be sufficient to predict Perf_100-km_ with an acceptable level of accuracy (SEE = 14.0%), especially in amateur athletes who want to perform a 100 km for the first time.
